# Characterization of the fecal microbiome during neonatal and early pediatric development in puppies

**DOI:** 10.1371/journal.pone.0175718

**Published:** 2017-04-27

**Authors:** Blake C. Guard, Hanna Mila, Jörg M. Steiner, Claire Mariani, Jan S. Suchodolski, Sylvie Chastant-Maillard

**Affiliations:** 1 Gastrointestinal Laboratory, Department of Small Animal Clinical Sciences, Texas A&M University, College Station, Texas, United States of America; 2 NeoCare, IHAP, Reproduction, Université de Toulouse, Toulouse, France; 3 Royal Canin, Aimargues, France; University of Minnesota, UNITED STATES

## Abstract

Limited information is available describing the development of the neonatal fecal microbiome in dogs. Feces from puppies were collected at 2, 21, 42, and 56 days after birth. Feces were also collected from the puppies’ mothers at a single time point within 24 hours after parturition. DNA was extracted from fecal samples and 454-pyrosequencing was used to profile 16S rRNA genes. Species richness continued to increase significantly from 2 days of age until 42 days of age in puppies. Furthermore, microbial communities clustered separately from each other at 2, 21, and 42 days of age. The microbial communities belonging to dams clustered separately from that of puppies at any given time point. Major phylogenetic changes were noted at all taxonomic levels with the most profound changes being a shift from primarily Firmicutes in puppies at 2 days of age to a co-dominance of Bacteroidetes, Fusobacteria, and Firmicutes by 21 days of age. Further studies are needed to elucidate the relationship between puppy microbiota development, physiological growth, neonatal survival, and morbidity.

## Introduction

The canine gastrointestinal (GI) microbiota is an integral factor for maintaining and promoting canine health [1–3]. It is now well established that in dogs and cats, the GI microbiota consortia typically consists of several hundred bacterial phylotypes [4–6]. Microbes in the GI tract are part of an intricate ecosystem which aid in the defense against invading pathogens, stimulate the immune system, aid in digestion, and extract energy for enterocytes [7]. Despite a rather extensive collection of research articles describing the gut microbiota and its relationship to GI disorders and metabolism [1, 6, 8–10], there are few data about GI microbiota composition in developing neonate puppies.

The microbiota of kittens have been described using DGGE (Denaturing Gradient Gel Electrophoresis) and this data suggests that microbial diversity and richness decreases from 4 weeks old through weaning [11]. A study in kittens using pyrosequencing where fecal samples were taken at 8, 12, and 16 weeks, however, show no significant changes in bacterial diversity among ages [12]. Studies in humans have investigated neonatal development and found that by three years of age, the microbial profile begins to appear adult-like [13–15]. Similar equine studies done in foals indicate that the fecal microbial community constantly changes until 30 days after birth. No changes were noted, however, at weaning (day 180) and the last sampling day (day 365) [16, 17]. Based on previous culture-based studies, the GI tract of beagle puppies was colonized at day 1 with anaerobic bacteria gradually increasing throughout post-natal development [18]. That same study reported decreased clostridia by day 21 whereas lactobacilli increased more than 100 fold between days 1 and 21. In addition, proportions of bacterial groups were found to be altered during significant life events such as weaning coupled with an evolving dietary regimen [18]. Denaturing gradient gel electrophoresis (DGGE) analysis of fecal samples from dogs of two different age groups (i.e., appx. 2.5 years old vs. 10 years old) showed only slight changes in bacterial banding patterns [19].

Appropriate development of neonate gut microbiota is likely essential for resistance to pathogens and decreased risk of neonatal mortality. Enteropathogens such as canine parvovirus and Giardia are frequent and serious health concerns for peri-weaning puppies, with clinical outcomes dependent on intestinal environment [20, 21]. Limited data is available that uses next-generation sequencing to evaluate the progression of puppy fecal microbiota in the early stages of life. Therefore, the aim of this study was to characterize the fecal microbiota from 2 to 56 days of age in growing puppies using 454-pyrosequencing. Our hypothesis was that the microbiota would undergo rapid changes in their microbial communities throughout the first few weeks of life.

## Materials and methods

### Animal enrollment and sample collection

The protocol was reviewed and approved by the Royal Canin Internal Ethics Committee (reference number: AF/20140704). Puppies and their mothers (n = 46 in total), belonging to ten different breeds (Table 1.–description of the population), were housed within one French breeding kennel. The mean age of the dams was 5 years old and the range was between 2 and 7 years old. These puppies were part of a randomized two arm study in which the puppies presented in this report served as the control. Since one week before until 2 months after parturition, dams were fed a dry balanced diet for growing dogs (Starter, Royal Canin, Aimargues, France) *ad libitum* (food composition: moisture 8%, protein 30%, crude fat 22%, crude fiber 1.8%, and ash 6.9%). Previously to being fed their starter diet, dams were fed Breeding Adult (Royal Canin) (food composition: moisture 9%, protein 22%, crude fat 18%, crude fiber 6.2%, and ash 4.5%) for a period of 7.5 to 9.5 months. Dams were allowed permanent access to food previous to and no antibiotics were administered to them during the duration of this study. All puppies were born by natural delivery (i.e., no cesarean-section). Fecal samples were collected by rectal swab from growing puppies (120 samples from 30 puppies) at 2, 21, 42, and 56 days after birth. Fecal samples from puppies were collected between 8 AM and 12 PM. A rectal swab was collected from each of the dams (n = 16) at a single time point within the first 24h after parturition. Feces from both dams and puppies were stored at -80°C immediately after collection until further processing. All puppies remained with their dams during the entire experiment and were allowed to suckle freely. Puppies were allowed to eat solid food (Starter) from 3 weeks of age, with progressive weaning until 8 weeks of age.

**Table 1:** Characteristics of puppies and bitches included in the study.

Breeds	Number of puppies	Mean birth weight (g)	Number of male puppies	Number of bitches
**Bichon Frise (S)**	2	234	1	1
**Bichon Maltese (S)**	4	176	3	2
**Cocker Spaniel (S)**	1	314	1	1
**Golden Retriever (L)**	5	336	2	1
**Jack Russel Terrier (S)**	2	241	0	0
**Labrador Retriever (L)**	3	431	1	3
**Lhassa Apso (S)**	5	183	4	3
**Poodle (S)**	5	195	2	3
**Shih Tzu (S)**	2	176	2	1
**West Highland White Terrier (S)**	1	230	1	1
**TOTAL**	30	247	17	16*

* Two samples from bitches were missing, their puppies were still included (1 from Golden Retriever and 1 from Jack Russel Terrier).

Breed size: S: Small (adult body weight < 10 kg); L: Large (adult body weight > 25 kg)

### DNA isolation

Approximately 100 mg of feces were aliquoted into a sterile 1.7 ml tube (Microtube, Sarstedt AG & Co, Nümbrecht, Germany) containing 150 μl of 0.1 mm zirconia-silica beads and 100 μl of 0.5 mm zirconia-silica beads (BioSpec Products Inc., Barlesville, OK, USA). Samples were then homogenized (FastPrep-24, MP Biomedicals, USA) for a duration of 1 minute at a speed of 4 m/s. DNA was then extracted with the ZR fecal DNA Mini Prep kit^™^ following the manufacturer’s instructions (Zymo Research, Irvine CA, USA).

### 454-pyrosequencing

Bacterial tag-encoded FLX-titanium amplicon pyrosequencing was performed using the primers 530F (5’-GTG CCA GCM GCN GCG G) and 1100R (5’-GGGTTN CGN TCG TTG) which were used to target the 600 base-pair region of the 16S rRNA genes [22]. Raw sequence data was screened, trimmed, de-noised, filtered, and depleted of chimeras using the QIIME (Quantitative Insights Into Microbial Ecology) open-source pipeline [23]. Operational taxonomic units (OTU) were assigned based on at least 97% sequence similarity using QIIME and open OTU picking was used. The sequences were deposited in the Sequence Read Archive under the following accession number: SRP055383.

### Quantitative PCR (qPCR)

Quantitative PCR was used as described previously [1, 24, 25]. Briefly, quantitative PCR reactions were performed using two reaction chemistries. For a subset of assays SYBR-green based reaction mixtures were used, with a total reaction volume of 10 μl. The final mix contained 5 μl SsoFast^™^ EvaGreen^®^ supermix (Bio-Rad Laboratories, CA, USA), 0.4 μl each of a forward and reverse primer (final concentration: 400 nM), 2.6 μl of high quality PCR water, and 2 μl of normalized DNA (final concentration: 5 ng/μl). Conditions for PCR were as follows: initial denaturation at 98°C for 2 min, then 40 cycles with denaturation at 98°C for 3 sec and annealing for 3 sec. Post-amplification, a melt curve analysis was performed using these conditions: 95°C for 1 min, 55°C for 1 min, and increasing incremental steps of 0.5°C for 80 cycles for 5 sec each. All samples were run in duplicate fashion. TaqMan^®^ based reaction mixtures were used in a total reaction volume of 10 μl. The final mix contained 5 μl TaqMan^®^ Fast Universal PCR master mix (Life Technologies, NY, USA), 0.4 μl of a forward and reverse primer (final concentration: 400 nM), 2 μl of high quality PCR water, and 2 μl of normalized DNA (final concentration: 5 ng/μl). Conditions for PCR were as follows: initial denaturation at 95°C for 20 sec then 40 cycles with denaturation at 95°C and annealing for 3 sec. Post-amplification, a melt curve analysis was performed using these conditions: 95°C for 1 min, 55°C for 1 min, and increasing incremental steps of 0.5°C for 80 cycles for 5 sec each. All samples were run in duplicate fashion.

### Statistical analysis

Data for all groups of dogs were tested for normality using a Shapiro-Wilk test. A non-parametric Kruskal-Wallis test blocking by individual to account for repeated measures or repeated measures ANOVA when three or more groups were compared at once was used where appropriate based on the distribution of data. In some case, repeated measures testing could only be carried out on a subset of puppies where sequencing data was available for all time points. A Dunn’s Post Test was then used to identify differences in groups where three or more comparisons were made, a p-value less than 0.05 was considered significant. When two groups were compared, a Mann-Whitney U test or t-test was used in accordance with how the data was distributed. When comparing individual bacterial groups in sequencing data, p-values were adjusted by Benjamini and Hochberg’s False Discovery Rate (FDR) to account for multiple comparisons, and a q<0.05 was considered significant. All statistical analyses were conducted using statistical software packages (JMP^®^ Pro version 10, SAS Institute Inc, Cary, NC and Prism version 5 Graph Pad Software Inc, La Jolla, CA). Microbial communities between groups were compared using the ANOSIM function (package PRIMER 6, PRIMER-E Ltd., Plymouth, UK) to evaluate beta diversity. Alpha diversity measures were found to be normally distributed therefore ANOVA was carried out to evaluate differences in observed species, chao1, Shannon diversity, and phylogenetic diversity (PD) whole tree indices. A post-test (i.e., Tukey’s multiple comparison test) was then used to elucidate individual differences between groups where appropriate.

The software PICRUSt (Phylogenetic Investigation of Communities by Reconstruction of Unobserved States) was used to make functional gene content predictions with quantifiable uncertainty based on 16S rRNA gene data present in the Greengenes database. PICRUSt is freely available online (https://huttenhower.sph.harvard.edu/galaxy/) in the Galaxy workflow framework and can also be used through the QIIME open-source pipeline [26].

The OTU table for input used in PICRUSt utilized closed OTU picking from the Green Genes 13_5 data files. All samples which met a cutoff of having 1400 sequences after OTU picking was performed using the Green Genes 13_5 data files per sample were included. The results in PICRUSt are represented on three levels, similar to the way in which taxonomic lineage is utilized: level 1 being the broadest category, level 2 being slightly more specific, and level 3 being the most specific to the KEGG orthologs (KO) that make up that gene family.

Linear discriminant analysis effect size (LEfSe) was used to elucidate taxa and genes associated with different periods during pre-weaning development. LEfSe is freely available online in the Galaxy workflow framework [27, 28]. Generally, an LDA score of greater than 2.0 is considered significant by default using LEfSe, however in some cases a higher LDA threshold was used to reduce the number of features to only the most significant identifying key changes in bacteria or KEGG pathways belonging to time points during pre-weaning development. LEfSe was used to generate cladograms to visualize these changes via the Galaxy workflow frame work mentioned previously.

## Results

### Sequencing summary

Analysis yielded 681,644 quality sequences for the 136 samples (mean ± standard deviation [of all samples] = 5012 ± 2763). The OTU table was rarified to 1400 sequences per sample to achieve equal sampling depth across all samples.

### Effect of age on beta diversity in growing puppies

**Fig 1:**
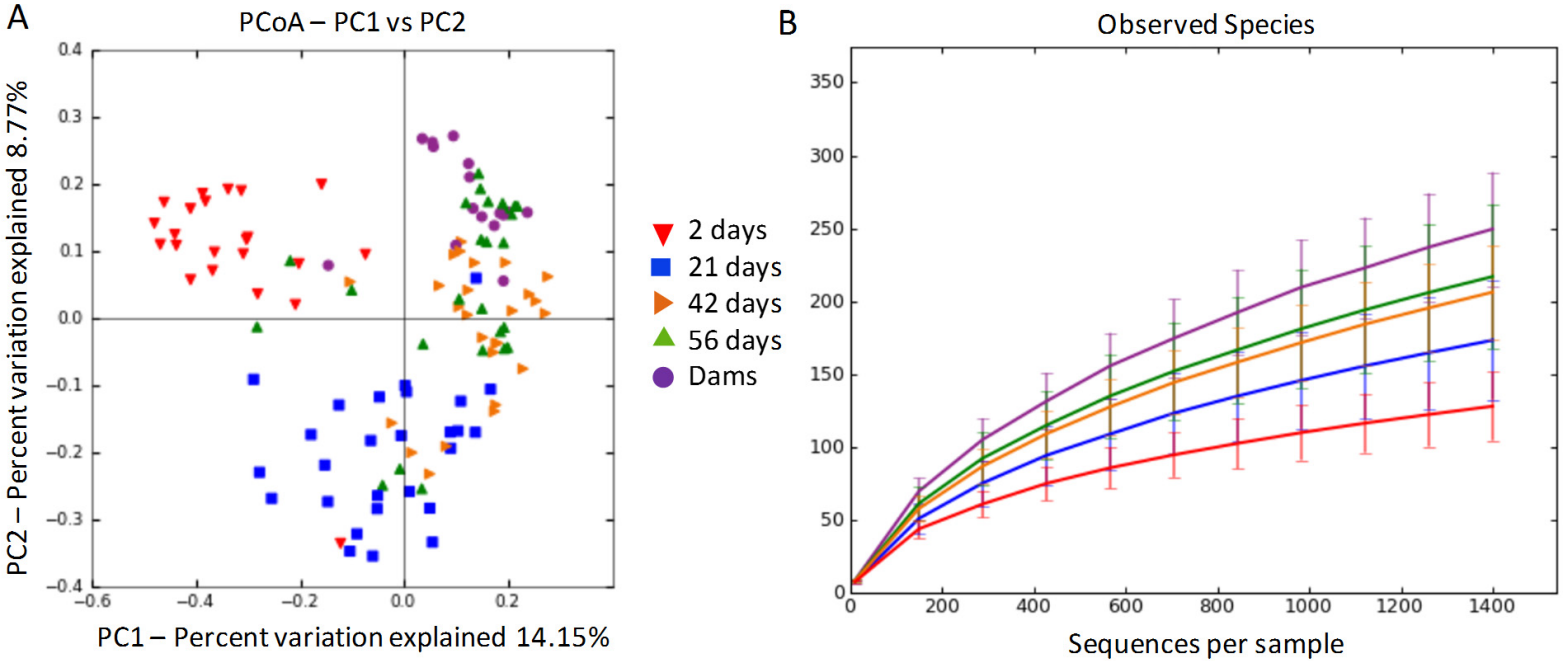
(Panel A): Unweighted UniFrac distances of 16S rRNA genes representing the difference in microbial communities (i.e., beta diversity) between dams and puppies during pre-weaning development. (Panel B): Observed species (i.e., alpha diversity) present in fecal samples belonging to dams and puppies during pre-weaning development. Up-side down red triangles represent puppies at 2 days old, blue squares represent puppies at 21 days old, sideways orange triangles represent puppies at 42 days old, right-side up green triangles represent puppies at 56 days old, and lastly purple circles represent the dams of the puppies. Colors of shapes also correspond with lines in Panel B which represent the mean accompanied by standard error bars.

Differences in microbial communities were assessed between all combinations of time points and dams. Microbial communities were found to cluster separately between 2, 21, and 42 days of age (ANOSIM: p < 0.001). In addition, the microbial communities belonging to puppies at 56 days of age clustered separately from their mothers (ANOSIM: p < 0.001). Table 2 summarizes comparisons with p-values and R statistic. Fig 1 (Panel A) depicts clustering in microbial communities (i.e., beta diversity) between puppies at all time-points and the dams.

**Table 2:** Comparison of microbial communities (i.e., beta diversity) during the development of pre-weaning puppies.

Group Comparison		R-Statistic	p-value
*Dams vs:	2 days	0.817	0.001
	21 days	0.601	0.001
	42 days	0.421	0.001
	56 days	0.136	0.033
**56 days vs**:	2 days	0.741	0.001
	21 days	0.388	0.001
	42 days	0.025	0.108
**42 days vs**:	2 days	0.883	0.001
	21 days	0.383	0.001
**2 days vs**.	21 days	0.729	0.001

*Dams were sampled at a single time point within 24 hours after parturition.

### Effect of age on alpha diversity in growing puppies

The time points were compared between every combination of 2, 21, 42, and 56 days as well as with the dams. For observed species, puppies at 21 and 42 days of age harbored significantly increased species richness compared to 2 days of age (ANOVA: p < 0.0001). Summary statistics for all alpha diversity metrics analyzed are provided in Table 3, and rarefaction curves representing the observed species in addition to sequencing depth are displayed in Fig 1 (Panel B).

**Table 3:** Summary statistics for species richness (i.e., alpha diversity measures) during pre-weaning development in growing puppies.

Alpha Diversity Metric	Days After Birth (mean^a^ ± standard deviation)					
	2	21	42	56	Dams	p-value
**Observed Species**	128 ± 24	173 ± 41	206^a^ ± 32	216^a^^,b^ ± 50	249^b^ ± 40	<0.0001
**Chao1**	214 ± 72	306^a^ ± 86	399^b^ ± 93	369^a^^,b^ ± 111	417^b^ ± 106	<0.0001
**Shannon Index**	4.8^a^ ± 0.5	5.1^a^ ± 0.7	5.6^b^ ± 0.5	5.8^b.c^ ± 0.6	6.2^c^ ± 0.5	<0.0001
**PD Whole Tree**	6.5^a^ ± 1.3	7.9^a^ ± 1.9	9.8^b^ ± 1.4	10.1^b^ ± 2.5	13.0 ± 1.7	<0.0001

^a^All means are significantly different than one another respective to alpha diversity measure unless their means share a common superscript with one another in which case those means are not significantly different than one another (p>0.05)

### Major phylogenetic differences during pre-weaning development

**Fig 2:**
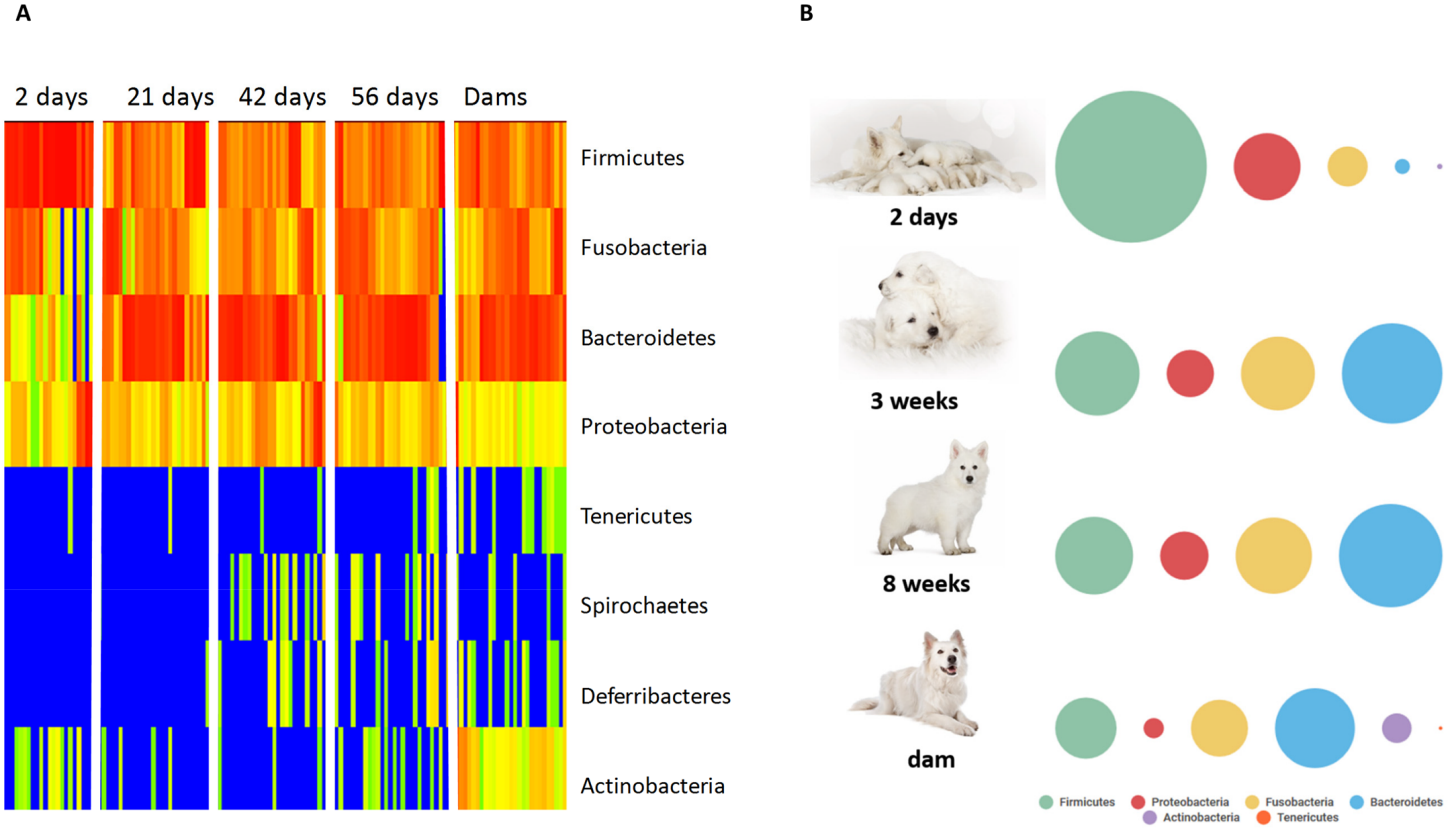
**(Panel A) Heatmap illustrating the relative abundance of predominant bacterial phyla in fecal samples belonging to dams and puppies during pre-weaning development**. Red, orange, and yellow colors represent increased abundance of indicated bacterial groups, while green and blue colors represent decreased abundance of indicated bacterial groups. **(Panel B) General changes in abundance at the phylum level based on a bubble plot**.

**Fig 3:**
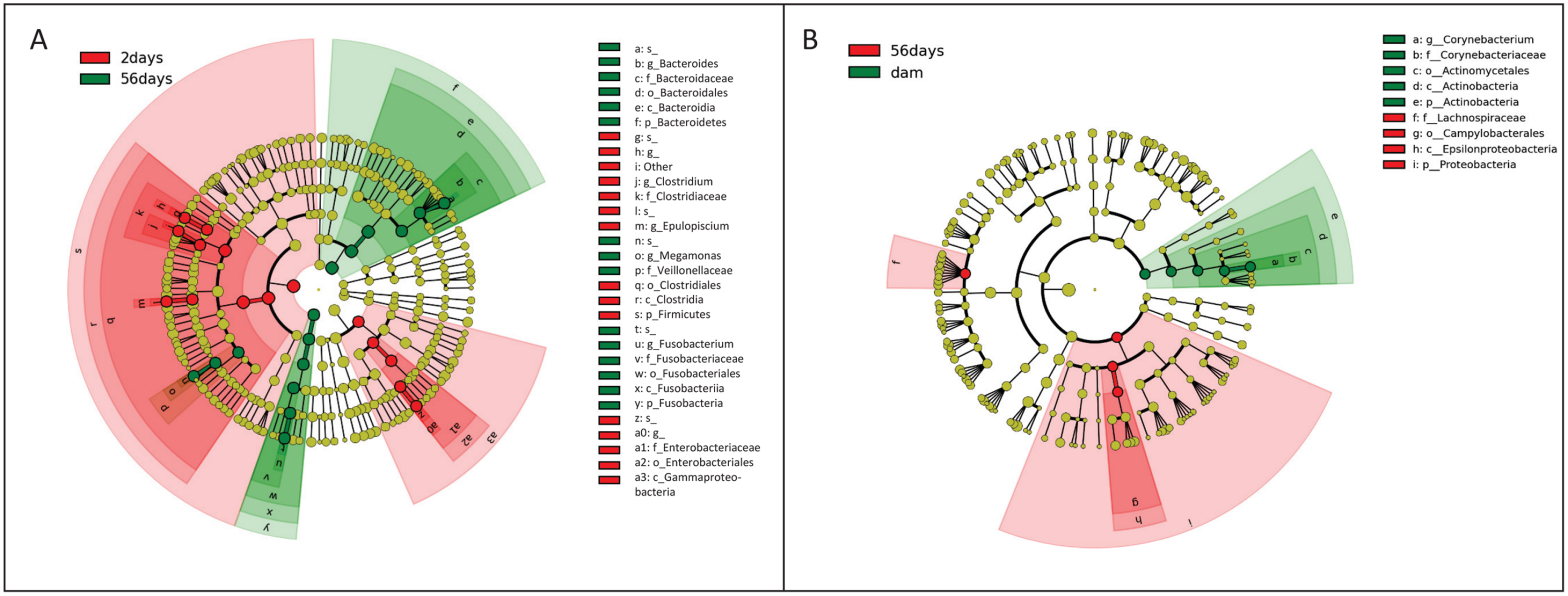
(Panel A): Cladogram describing differentially abundant bacteria between 2 and 56 days after birth in puppies, significant features based on LDA threshold of > 4.5. (Panel B): Cladogram describing differentially abundant bacteria between puppies at 56 days of age and their mothers, significant features based on LDA threshold of > 3.5. Names of bacteria which follow a p, c, o, f, g, s belong to the respective phylum, class, order, family, genus, or species. For classifiers such as s__ and g__ which do not have a bacterial name following are species that are unclassified based on the 16S rRNA gene data. Figure generated using LEfSe.

Analysis of bacterial groups using univariate repeated measures testing showed that 5 out of 8 phyla, 12 out of 15 classes, 18 out of 23 orders, 27 out of 52 families, 48 out of 109 genera, and 56 out of 137 species were identified as having a significant difference between time points (q<0.05). On the phylum level, the percent of sequences belonging to Firmicutes dominated the fecal microbiota at 2 days of age (median percent of sequences = 64.3) compared with days 21, 42, and 56 (median percent of sequences = 26.11, 22.25, and 22.29, respectively; q<0.0001). At two days old, Bacteroidetes was less than one percent of all sequences, however by day 21, 42, and 56 there was a significant increase to approximately 37% of all sequences (q<0.0001). Fusobacteria and Proteobacteria were other prominent members of the fecal microbiota comprising approximately 16% and 11%, respectively, of sequences in all time points. Fig 2 shows a heatmap and bubble plot (panel A and B, respectively) illustrating changes at the phylum level in growing puppies and their mothers. S1 Table contains summary statistics for all taxonomic classifications (i.e., phylum, class, order, family, genus, and species). LEfSe was used to identify global changes in the percent of sequences belonging to any level of bacterial group between puppies at 2 days old and puppies at 56 days old. An LDA threshold of 4.5 was used. Fig 3 (Panel A) shows a cladogram with differentially abundant taxa.

### Major phylogenetic changes between puppies at 56 days of age and their mothers

Univariate analysis identified 3 out of 8 phyla, 5 out of 15 classes, 7 out of 23 orders, 4 out of 49 families, 2 out of 96 genera, and 3 out of 123 species as having a significant difference in the percent of sequences belonging to either the puppies at 56 days of age or the dams (q<0.05). On the phylum level, the three most abundant phyla for both dams and their puppies at 56 days of age were Bacteroidetes, Firmicutes, and Fusobacteria. Sequences were significantly increased in the dams compared to the puppies at 56 days of age belonging to Actinobacteria (median percent of sequences = 5.92 and 0.00 respectively; q<0.0001) and Tenericutes (median percent of sequences = 0.07 and 0.00 respectively; q<0.0107). Proteobacteria was significantly decreased in dams compared to the puppies at 56 days old (median percent of sequences = 2.71 and 8.67 respectively; q<0.0005). S2 Table contains summary statistics for all taxonomic classifications belonging to only puppies at day 56 and the dams (i.e., phylum, class, order, family, genus, and species). LEfSe was used to identify global changes in the percent of sequences belonging to any level of bacterial group between puppies at 56 days of age and their mothers. An LDA threshold of 3.0 was used. Fig 3 (Panel B) shows a cladogram with differentially abundant taxa.

### Quantitative polymerase chain reaction (qPCR) for select bacterial groups

**Fig 4:**
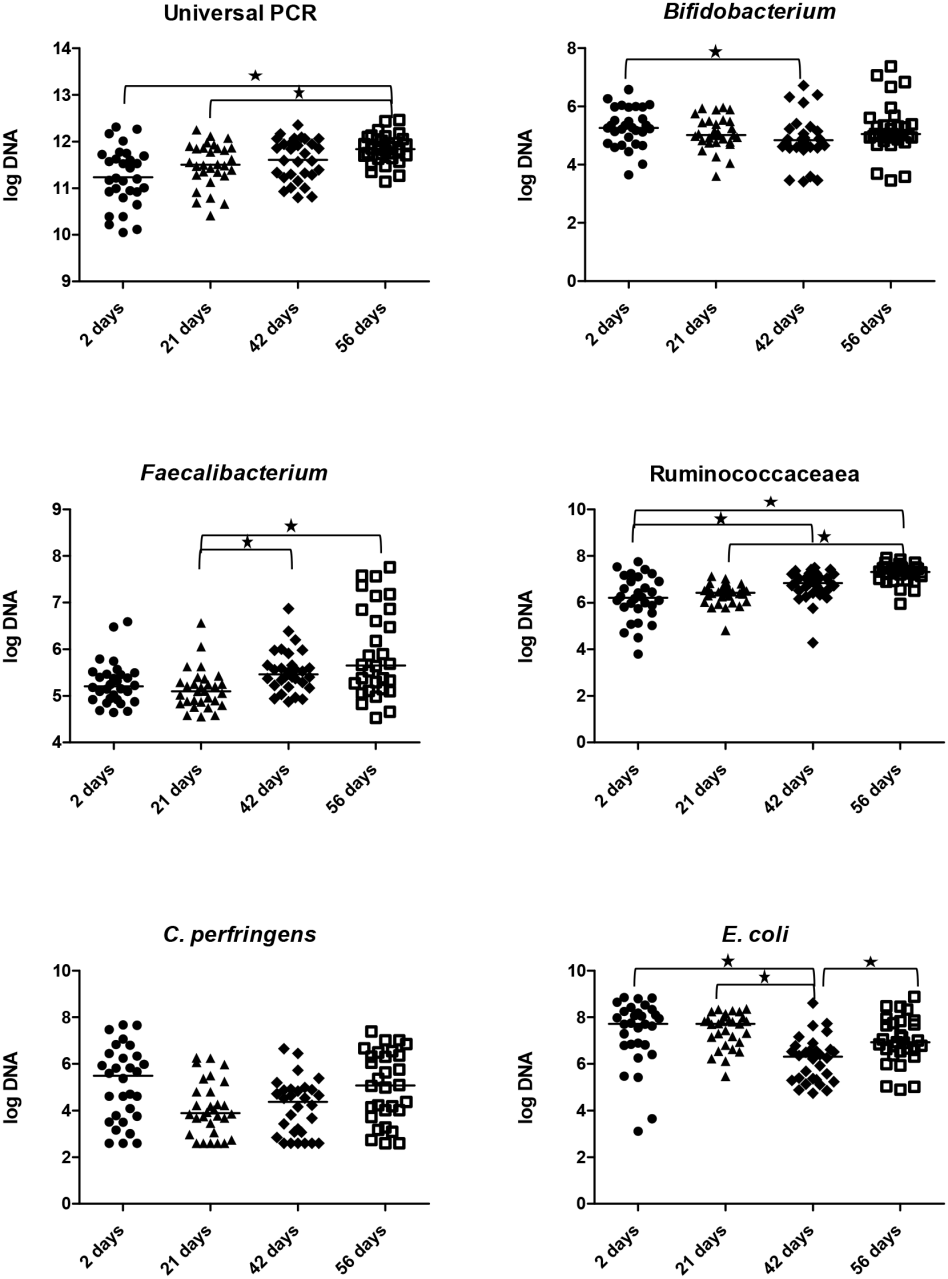
Quantitative PCR for select bacterial groups during pre-weaning development in puppies. A star indicates a Dunn’s post-test significance of p < 0.05 between bracketed groups.

To validate the sequencing results and to detect groups that are generally underrepresented in sequencing data, qPCR was used. Fig 4 illustrates the difference in relative abundance between time points and the bacterial groups (i.e., universal 16S, *Bifidobacterium*, *Escherichia coli*, *Faecalibacterium*, *Ruminococcaceae*, *Clostridum perfringens*) investigated by qPCR. The total amount of bacteria measured in feces was significantly increased (p = 0.0008, Dunn’s post-test p<0.05) in puppies at day 56 (log DNA median [min-max]: 11.8 [11.1–12.4) compared with puppies at days 2 (log DNA median [min-max]: 11.2 [10.0–12.3) and 21 (log DNA median [min-max]: 11.5 [10.4–12.2). The relative abundance of Ruminococcaceae was significantly increased (p<0.0001, Dunn’s post-test p<0.05) in puppies at day 56 (log DNA median [min-max]: 7.3 [5.9–7.9) compared with puppies at day 2 (log DNA median [min-max]: 6.2 [3.7–7.7]). While all of these comparisons upon Friedman’s testing reached statistical significance, there was not always a clear pattern in bacterial abundances upon Dunn’s Post-Testing. Those that have been reported here displayed clear increases over the time points analyzed. A complete summary statistic table has been included in S3 Table.

### The effect of litter, antibiotics, and breed size in growing puppies on microbiota

Confounding factors such as the potential effect of littermates, antibiotic administration, and breed size were assessed. Unfortunately, the number of puppies in this study belonging to the same mother was too few to make noteworthy comparisons with regards to differences in microbial communities based on littermates. No differences were observed in microbial communities between those puppies that had or had not received antibiotics. A significant difference in microbial communities between large and small breed puppies at day 42, however, was noted (ANOSIM p = 0.0200, R-Statistic: 0.3254). S1–S4 Figs contain microbial communities and beta diversity representations of these comparisons. Diarrhea developed at some point in time throughout the study in 16 of the 30 puppies analyzed. Unfortunately, the duration and time in which diarrhea occurred was sporadic which made these statistical comparisons difficult to conduct.

**Fig 5:**
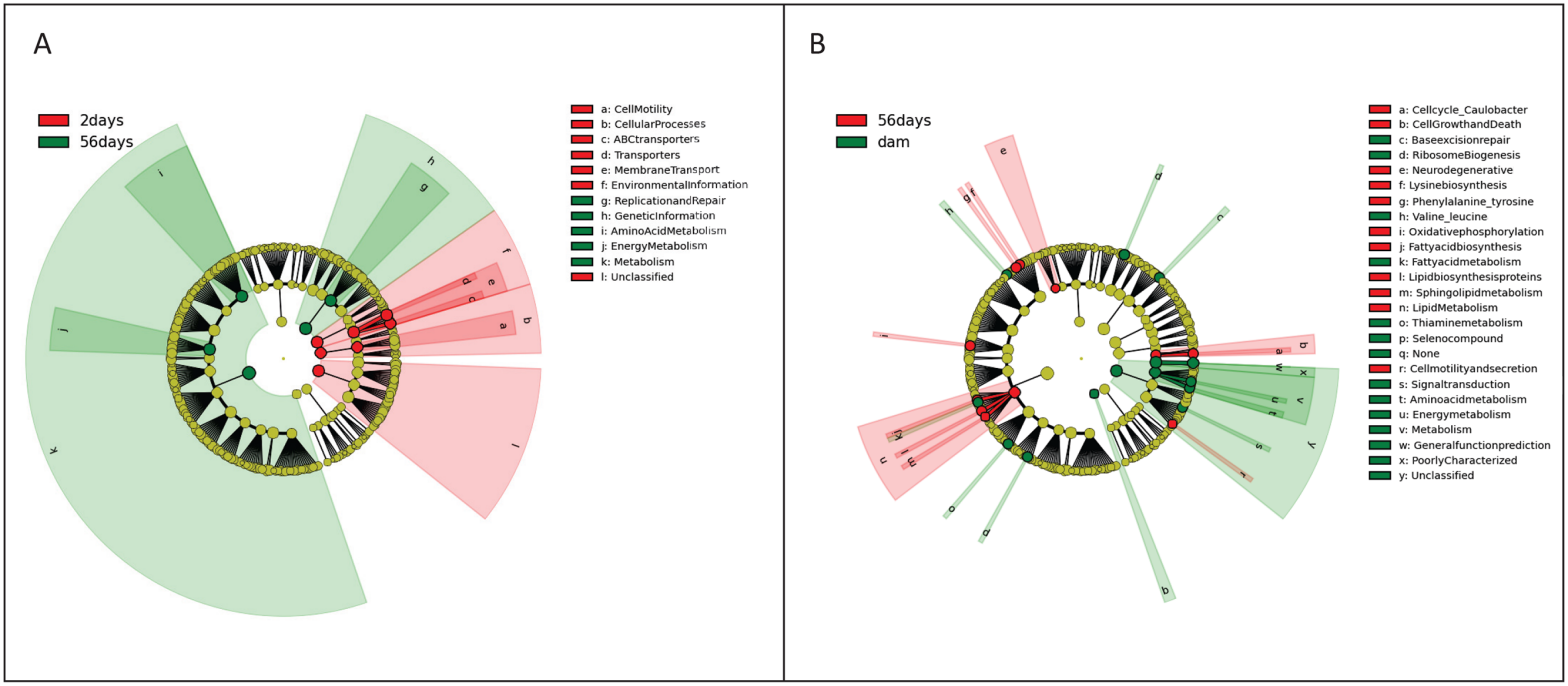
(Panel A): Cladogram describing differentially abundant functional gene families between 2 and 56 days after birth in puppies, significant features based on LDA threshold of > 3.5. (Panel B): Cladogram describing differentially abundant bacteria between puppies at 56 days of age and their mothers, significant features based on LDA threshold of > 2.0.

Functional gene profiles generated by PICRUSt representing neonatal development in puppies and the dams analyzed by LEfSe: Fig 5 (Panel A) shows differentially abundant features between the first and last time point in puppies. An LDA threshold of 3.5 or more was considered highly significant. Puppies that were 2 days of age harbored significantly increased KO compared to puppies at 56 days of age belonging to Environmental Information Processing, Cellular Processes, Unclassified, Membrane Transport, Cell Motility, and Transporters/ABC Transporters. While puppies at 56 days of age harbored significantly increased KO compared to puppies at 2 days of age belonging to Metabolism, Genetic Information Processing, Replication/Repair, Amino Acid Metabolism, and Energy Metabolism. Fig 5 (Panel B) shows differentially abundant features between puppies at 56 days of age and their mothers. An LDA threshold of 2.0 or more was considered highly significant. Puppies that were 56 days of age harbored significantly increased KO compared to their mothers belonging to lipid metabolism, lysine biosynthesis, oxidative phosphorylation, fatty acid biosynthesis, phenylalanine/tyrosine/tryptophan biosynthesis, sphingolipid metabolism, as well as lipid biosynthesis proteins. The dams harbored significantly increased KO compared to puppies at 56 days of age belonging to thiamine metabolism, fatty acid metabolism, valine/leucine/isoleucine degradation, and seleno compound metabolism. S4 Table contains summary statistics for PICRUSt results expressed as a percent of total KO output for each time point belonging to the puppies and the dams.

## Discussion

This study describes and characterizes the fecal microbial profile of puppies from 2 days, 21 days, 42 days, and 56 days post-natal development. Solid food was introduced at 3 weeks with progressive weaning thereafter in growing puppies. Significant shifts were observed in microbial communities belonging to puppies during pre-weaning development. These results show that by 8 weeks after birth, the fecal microbiome in puppies is still significantly different from that of their mothers. Contribution of the microbiota of the mother to the puppies’ microbiota remains to be determined. One of the original goals of this study was to be able to evaluate the effect of litter on the fecal microbiome. But as we addressed in the results section, we were unable to get a significant number of samples to achieve satisfactory statistical power. It may be that the feces of dams that have recently given birth may not be entirely representative of that of a healthy fecal microbiome given the stressful event of parturition. However, other cases have been noted in healthy dogs where similar phyla co-dominate the fecal microbiota (i.e., Bacteroidetes, Firmicutes, and Fusobacteria). This trend is noticed in puppies as they reach 8 weeks of age seen elsewhere [6]. Given the resources available for this study it seemed most appropriate use the dams as a reference for the fecal microbiota in adult dogs to compare to puppies as they aged, while controlling for sample collection, primer design, and sequencing platform.

There are a variety of studies in human neonates where the microbiota has been assessed. One case study investigated the fecal microbiota in an infant for a period of 2.5 years from birth through 60 fecal samples [29]. Their findings suggested an increase in phylogenetic diversity over time which was positively correlated with age (R^2^ = 0.5) and dietary changes during development. This is a difficult correlation to make in puppies since weaning in puppies can mean the transition from suckling milk to consuming solid food, which occurs between 4 and 6 weeks for most puppies. In some cases, the consumption of solid food can start as early as 3 weeks (e.g., commonly seen in large breed dogs) and for others only 5–6 weeks (e.g., mostly seen in small breed dogs). Weaning by definition can also represent a separation from the dam, which presents the moment when puppies have no more access to milk. In the present study, puppies at 21 days old were considered to still be drinking milk, by 42 days it varied among breeds, but by 56 days all puppies were eating almost exclusively solid food. In our study a significant difference in intestinal microbiota between small and large breed puppies was determined only at 42 days of age. Indeed, at that moment a dietary difference could be observed between these two breed sizes, probably explaining the different microbial communities observed in the fecal samples.

Observing the development of the canine neonate microbiome gives insight into opportunistic microbes which settle early on in the gut. Results from this study indicate *Clostridium* (belonging to the phylum Firmicutes) accounted for almost 10% of sequences identified in puppies at day 2 compared to approximately 1% of sequences identified at each time point thereafter. Other members of the family Clostridiaceae were also found to be prominent at day 2 and then decreased over time. This finding is in agreement with the distal luminal contents of neonatal beagles where clostridia were found to decrease only after 21 days of age [18]. Clostridial species are highly diverse. For example, *Clostridium* clusters XIVa and IV are largely involved in the release of butyrate as an energy source for colonocytes and serve a beneficial role in the gastrointestinal tract [30]. In contrast, *Clostridium perfringens* have long been associated with enteric pathogens affecting a multitude of animal species [31]. The early colonization of bacteria in the gut is crucial in establishing resistance to invading pathogens [32]. It would be useful to investigate the role of Clostridial groups by means of isolates in canine models to describe the functional impact of bacterial alterations in the growing puppy. Additionally, determination of species associated with a higher risk of neonatal pathologies would be of interest in order to control the high morbidity and mortality rate in pre-weaning puppies. Finally, studies in canine newborns could contribute to a better understanding of pathogenesis of some neonatal issues (e.g., necrotizing enterocolitis in preterm babies).

*Escherichia coli* was found to be significantly increased in puppies at 2 and 21 days compared to 42 days after birth according to qPCR data and was mirrored by sequencing data. Studies in human infants have shown that *E*. *coli* appears in the feces just a few days after birth [33, 34]. The cause of this finding could be due to the exposure of mothers feces and the infant upon delivery [35]. The decrease in the relative abundance of *E*. *coli* over the period of several weeks may be indicative of the transition from an aerobic to a sufficiently anaerobic environment. Certain strains of *E*. *coli* may provide a benefit to the host as shown in studies where babies, dosed orally with nonpathogenic *E*. *coli*, harbored less antibiotic-resistant *E*. *coli* strains than controls [36, 37].

Promoting the development of beneficial bacteria may be of interest in neonates. Buddington et al. (2003) described a 100- fold increase in *Lactobacilli* by day 21 in the luminal contents of the distal colon in neonate beagle puppies. Human breast milk has been shown to be a source of lactic acid bacteria in the infant gut [38] and canine milk reportedly can be a natural source of lactobacilli for the suckling puppy [39]. The present study found that less than one percent of all sequences in the feces of puppies at any day evaluated belonged to *Lactobacillus*. It would be useful to validate *Lactobacillus* sequencing results with qPCR and potentially identify any alterations in the bacterium during development, unfortunately, there was insufficient quantity of sample left to perform these tests.

**Fig 6:**
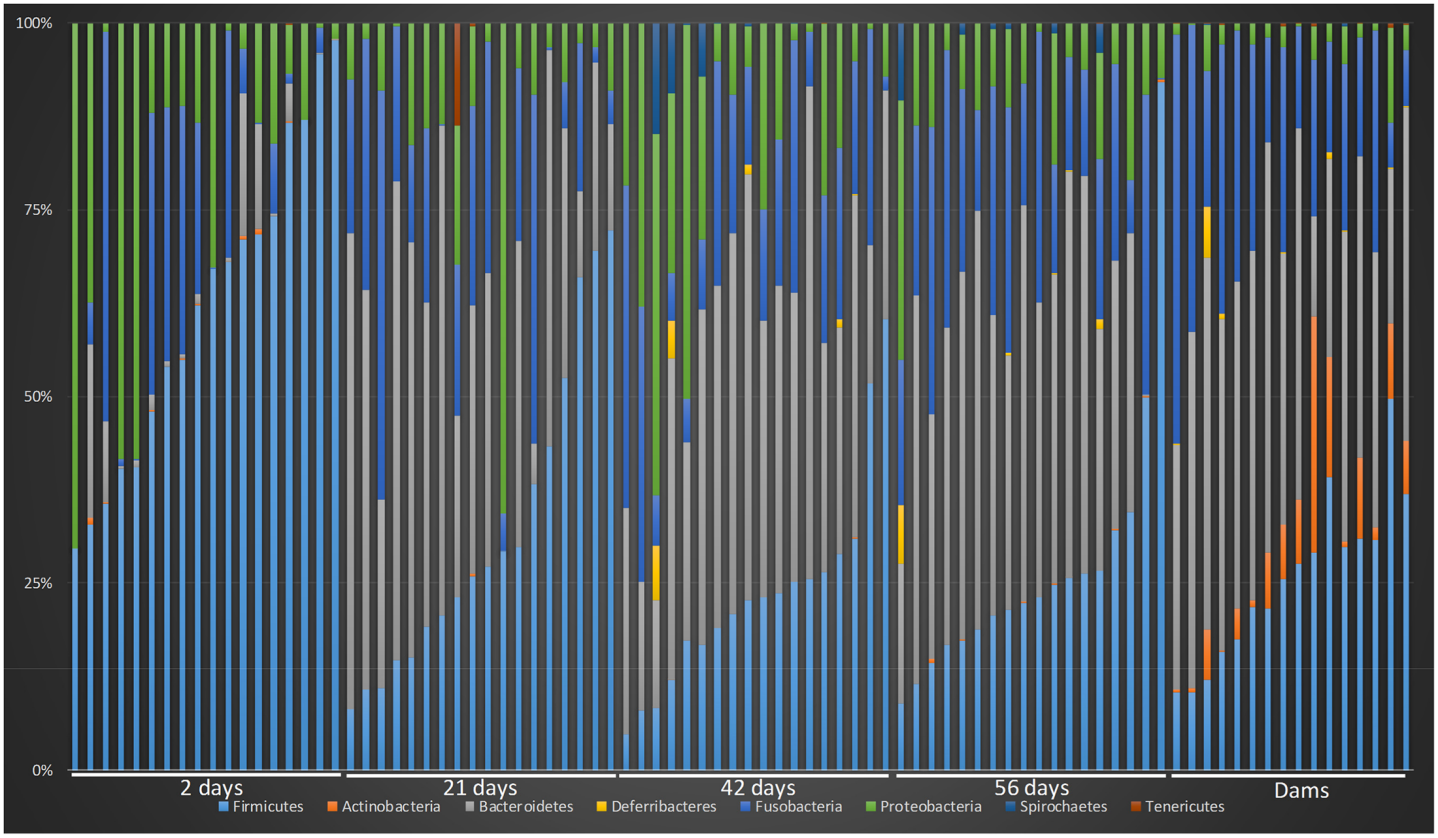
Inter-individual variability based on the percent of sequences belonging to phylum level taxa during pre-weaning development along with their mothers. Each individual bar represents the distribution of bacterial belonging to each phyla for a particular puppy or dam.

Studies have suggested that the temporal variability during development of the human infant intestinal microbiota at the phylum level was higher in the infant than that of adult intestinal microbiota [40]. Despite this high variability, core bacterial groups such as Proteobacteria, *Bacteroides*, Firmicutes, Actinobacteria, and Verrucomicrobia, were present in both the infant and adult microbiota. By approximately 6 months of life, the human intestinal microbiota becomes dominated by *Bacteroides* and Firmicutes, which begins to shape the generic adult-like profile. In the present study, the variability of these major bacterial phyla was high with a range of 29–95% of sequences belonging to Firmicutes in 2 day old puppies against 10–35% of the sequences in the dams (Fig 6). By the time puppies had reached 56 days of age, their fecal microbiota represented a greater diversity of phylum level bacteria which was predominated by Bacteroidetes, Firmicutes, Fusobacteria, and Proteobacteria (median percent of sequences: 39, 22, 21, and 8, respectively). Their mothers harbored Bacteroidetes, Firmicutes, Fusobacteria, Actinobacteria, and Proteobacteria (median percent of sequences: 42, 25, 21, 5, and 2, respectively).

The observed convergence of highly variable bacterial profiles in neonate puppies that becomes more stable in adult life may be expected. The healthy mammalian microbiome shows considerable variability. However, age and disease processes generally overshadows healthy individual differences [41]. For instance, despite variability in the healthy human microbiota among individuals, it has been reported that a particular individual’s microbiota will remain stable overtime [42]. Faith et al. report that 60% of strains remained stable for over the course of 5 years in 37 healthy adults. Extrapolation of their data suggested that certain GI strains are residents for decades. Early colonizers may have thus a very important role on the development of mammalian gut microbiota. A combination of parental, environmental, dietary, and pathogen exposure may shape the development of the gut microbiota and thus provide a basis for unique mammalian microbial profiles.

Another factor that likely plays a role in early colonization of the mammalian gut is varying propensities and selectivity for bacteria based on their respiration preference. In human neonates, meconium, forming the first feces, is generally predominated by Firmicutes [43]. The majority of microbes consisted of Bacilli which are mainly composed of obligate or facultative aerobes. At 2 days of age in the present study, Firmicutes were also dominant (64%), however, the makeup of these were approximately 5% Bacilli and 58% Clostridia (which are known for being anaerobic). The differences may be explained by the rapid evolution of the gut that occurs over 2 days after birth. By 21 days, the greatest changes are found at the phylum level, whereby *Bacteroides* predominate the GI tract (i.e., median percent of sequences increases from <1% to approximately 30% in each subsequent time point). *Bacteroides* (a part of the phylum Bacteroidetes) then begin to establish themselves as strict anaerobes in their newly colonized environment, much like what has been reported in the mature human GI tract [44].

Inferred metagenomic analysis performed using PICRUSt identified an increased presence of genes belonging to cellular motility, cellular processes, and ABC transporters at 2 days of age compared to day 56 in puppies. The meconium at day 3 in human infants revealed an enrichment of ABC transporters which has been linked to antibiotic resistance [29]. The same study reported that that genes coding for cellular components were enriched as early as day 6, like found in the feces of puppies by 2 days after birth. The early on appearance of functional genes related to cellular components suggests the development of the innate immune system and development of the gut epithelium integrity.

By 56 days compared to 2 days after birth, puppies began to harbor differentially abundant genes responsible for metabolism including amino acid metabolism and energy metabolism. By 56 days, puppies were weaned (i.e., did not receive milk) and were maintained on a solid food source. Their microbiota became co-dominated by Bacteroidetes, Fusobacteria, and Firmicutes which are likely to help contribute to aiding in the digestion and utilization of macronutrients. Studies have shown this to be beneficial for the generation and maintenance of the immune system [45]. The increased number of genes belonging to amino acid metabolism may aid in T-cell function facilitating proper immune responses [46], and furthermore, may be explained by introduction to synthesized dietary regimens.

Sample size may be considered a limitation of this study as with different and sometimes infrequent disease pathologies, statistical comparisons can be hard to accurately make while still drawing meaningful conclusions. This could add useful insight into microbial changes that relate to pathological occurrences. Increased sample size may also help solidify whether true differences are present between large and small breed dogs and if these microbial communities converge after 42 days or if they may continue to develop into unique microbial profiles. Additionally, the fecal microbiome of the dams during parturition may not be completely representative of their normal microbiota. Further studies, should consider collecting fecal samples before and well after parturition. In general, however, we do not believe that this had any major effects on the healthy fecal microbiome.

## Conclusion

In conclusion, the results from this study indicate clear shifts in the fecal microbiota in puppies from 2 to 56 days after birth. These shifts are characterized by increased microbial diversity as well as species richness. Increased species richness may be indicative of the gastrointestinal tract of puppies gaining strength and resiliency against environmental threats. By day 42, microbial communities (i.e., beta diversity) appeared to have reached a relative stability. Differences were still apparent between puppies and their mothers by 56 days of age based on univariate analysis, however they were less obvious. It is likely that communities were still continuing to diversify by 56 days, but major shifts had already taken place. Factors driving the implantation of the gut microbiota should furthermore be considered (i.e., vaginal, oral, milk-borne, and fecal sources from the mother). Subsequently these findings may be valuable in establishing the most advantageous GI microbiota while simultaneously establishing a profile with resistance to enteropathogens during early microbial orientation in puppies.

## Supporting information

S1 Fig (Panel A): Principal Coordinate Analysis of unweighted UniFrac distances of 16S rRNA genes representing the difference in microbial communities (i.e., beta diversity) between large breed (red circles) and small breed dogs (blue squares) at 2 days of age. (Panel B): Principal Coordinate Analysis of unweighted UniFrac distances of 16S rRNA genes representing the difference in microbial communities (i.e., beta diversity) between no future antibiotic administration (red circles) and future antibiotic administration (blue squares) at 2 days of age.
(PDF)

S2 Fig (Panel A): Principal Coordinate Analysis of unweighted UniFrac distances of 16S rRNA genes representing the difference in microbial communities (i.e., beta diversity) between large breed (red circles) and small breed dogs (blue squares) at 21 days of age. (Panel B): Principal Coordinate Analysis of unweighted UniFrac distances of 16S rRNA genes representing the difference in microbial communities (i.e., beta diversity) between no antibiotic administration (red circles) and antibiotic administration (blue squares) before 21 days of age.
(PDF)

S3 Fig (Panel A): Principal Coordinate Analysis of unweighted UniFrac distances of 16S rRNA genes representing the difference in microbial communities (i.e., beta diversity) between large breed (red circles) and small breed dogs (blue squares) at 42 days of age. (Panel B): Principal Coordinate Analysis of unweighted UniFrac distances of 16S rRNA genes representing the difference in microbial communities (i.e., beta diversity) between no antibiotic administration (red circles) and antibiotic administration (blue squares) at any point in time during the study at 42 days of age.
(PDF)

S4 Fig (Panel A): Principal Coordinate Analysis of unweighted UniFrac distances of 16S rRNA genes representing the difference in microbial communities (i.e., beta diversity) between large breed (red circles) and small breed dogs (blue squares) at 56 days of age. (Panel B): Principal Coordinate Analysis of unweighted UniFrac distances of 16S rRNA genes representing the difference in microbial communities (i.e., beta diversity) between no antibiotic administration (red circles) and antibiotic administration (blue squares) at any point in time during the study at 56 days of age.
(PDF)

S1 Table Summary statistics of sequencing data describing the median percent of sequences belonging to puppies during neonatal development. (XLSX)

S2 Table Summary statistics of sequencing data describing the median percent of sequences belonging to puppies at 56 days of age and the dams. (XLSX)

S3 Table Quantitative PCR results for select bacterial groups. (XLSX)

S4 Table Summary Statistics of PICRUSt data expressed as a percent of total KEGG orthologs for puppies during neonatal development and the dams. (XLSX)
