# Supplementary material for: Characterization of the fecal microbiome during neonatal and early pediatric development in puppies
Source: PLoS One. 2017 Apr 27;12(4):e0175718. doi: 10.1371/journal.pone.0175718 (PMC5407640; doi:10.1371/journal.pone.0175718)
Supplement: S2 Fig — (Panel A): Principal Coordinate Analysis of unweighted UniFrac distances of 16S rRNA genes representing the difference in microbial communities (i.e., beta diversity) between large breed (red circles) and small breed dogs (blue squares) at 21 days of age. (Panel B): Principal Coordinate Analysis of unweighted UniFrac distances of 16S rRNA genes representing the difference in microbial communities (i.e., beta diversity) between no antibiotic administration (red circles) and antibiotic administration (blue squares) before 21 days of age. (PDF) [file pone.0175718.s002.pdf]

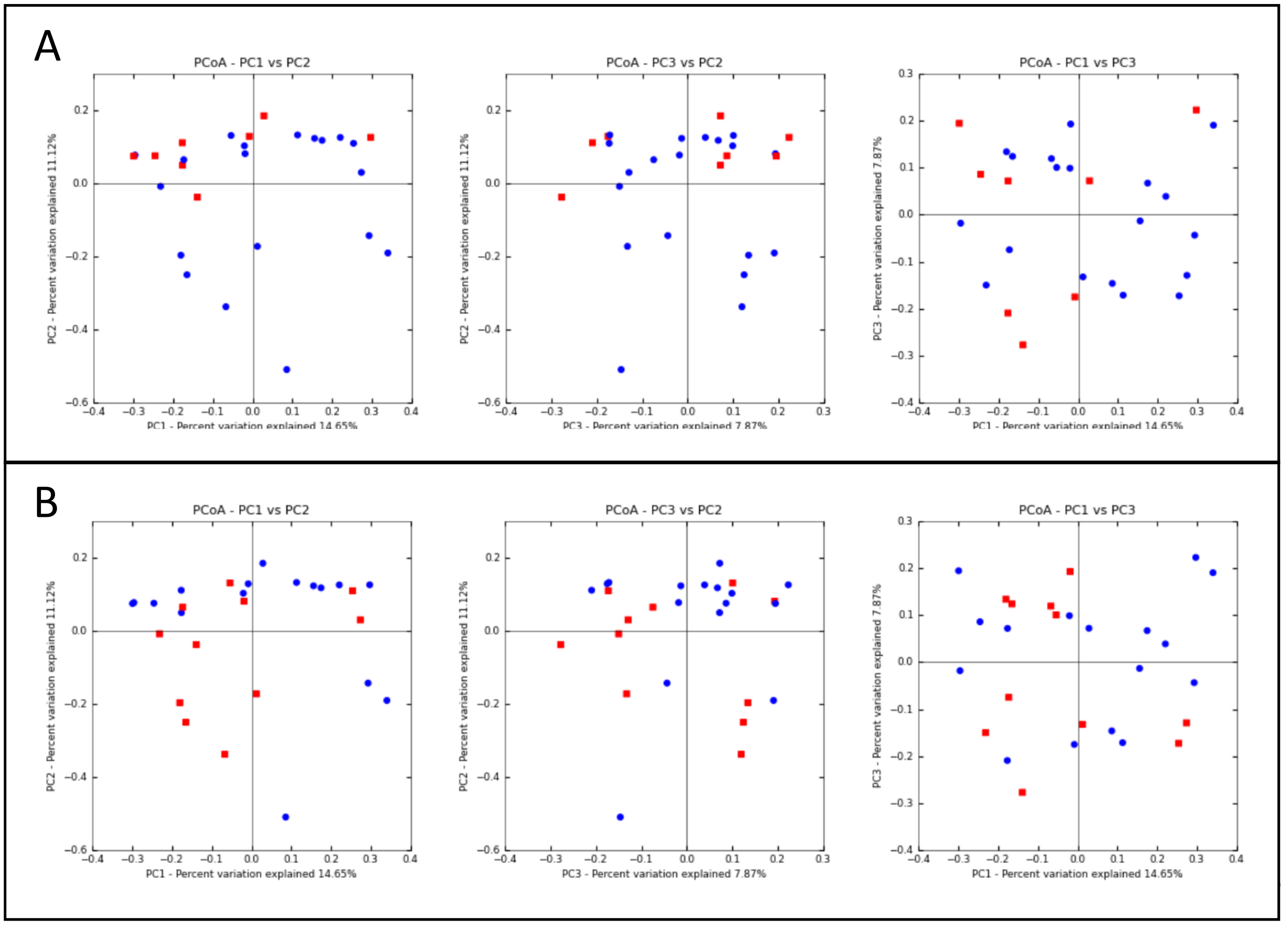

S2 Fig. (Panel A): Principal Coordinate Analysis of unweighted UniFrac distances of 16S rRNA genes representing the difference in microbial communities (i.e., beta diversity) between large breed (red circles) and small breed dogs (blue squares) at 21 days of age. (Panel B): Principal Coordinate Analysis of unweighted UniFrac distances of 16S rRNA genes representing the difference in microbial communities (i.e., beta diversity) between no antibiotic administration (red circles) and antibiotic administration (blue squares) before 21 days of age.
